# Spontaneous rupture of nontraumatic hepatic artery pseudoaneurysm

**DOI:** 10.1093/jscr/rjad201

**Published:** 2023-04-22

**Authors:** Andrea Boyd-Tressler, Craig Follette, Kelly Oechsel, Samuel Carmichael, Gregory R Stettler, Martin Avery

**Affiliations:** Department of Surgery, Atrium Health Wake Forest Baptist Hospital, Winston-Salem, NC, USA; Department of Surgery, Division of Trauma and Acute Care Surgery, Atrium Health Wake Forest Baptist Hospital, Winston-Salem, NC, USA; Wake Forest School of Medicine, Winston-Salem, NC, USA; Department of Surgery, Division of Trauma and Acute Care Surgery, Atrium Health Wake Forest Baptist Hospital, Winston-Salem, NC, USA; Department of Surgery, Division of Trauma and Acute Care Surgery, Atrium Health Wake Forest Baptist Hospital, Winston-Salem, NC, USA; Department of Surgery, Division of Trauma and Acute Care Surgery, Atrium Health Wake Forest Baptist Hospital, Winston-Salem, NC, USA

## Abstract

Spontaneous rupture of hepatic artery pseudoaneurysms (HAP) is a rare cause of intra-abdominal hemorrhage. Herein, we present a case of a spontaneous rupture of a nontraumatic HAP. A 61-year-old female, not on any anticoagulant or antiplatelet medications, presented with abdominal pain and hemorrhagic shock. Cross-sectional imaging revealed a left HAP with evidence of active bleeding. Emergent diagnostic angiography was performed, and angioembolization of an actively bleeding pseudoaneurysm was performed. Given the risk of rupture and high mortality rate associated with rupture, aggressive treatment of HAP should be pursued.

## INTRODUCTION

The differential diagnosis of intra-abdominal hemorrhage is broad and can include traumatic injury, complications of anticoagulant therapy, bleeding neoplasia or rupture of visceral aneurysms. Hepatic artery aneurysms (HAA) and pseudoaneurysms are rare with the true incidence being unknown [1, 2]. However, over the last several years, the identification of this entity has increased, likely secondary to improved imaging technology and the increased use of minimally invasive percutaneous biliary procedures, which can cause iatrogenic injury. Several risk factors for hepatic artery pseudoaneurysms (HAP) exist and include iatrogenic injury from instrumentation during open, endoscopic or Interventional Radiology (IR) procedures, development following liver transplantation, as sequela of blunt or penetrating abdominal trauma, and inflammatory or infectious conditions [3, 4]. HAP have a high rupture risk compared with other visceral artery aneurysms (VAAs) [3]. Mortality from HAP rupture is estimated to be between 20 and 44%; therefore, aggressive treatment rather than observation is warranted [3, 5, 6]. Herein, we present a case of a spontaneous rupture of an HAP and treatment with angioembolization.

## CASE REPORT

**Figure 1:**
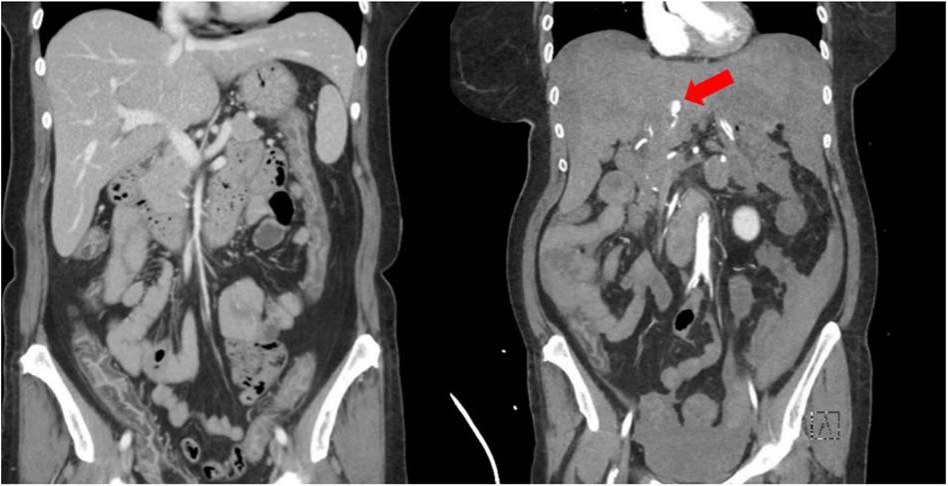
Imaging showing initial CT scan (left) and repeat CT angiogram (right) several hours later. Red arrow is identifying left HAP.

A 61-year-old female with a past medical history of hyperlipidemia, hypertension and diabetes presented to a referring facility emergency department with abdominal pain, nausea, vomiting, diarrhea and headache for 2 days. The patient did not take anticoagulants or antiplatelet therapy. She had no history of trauma. A computed tomography (CT) scan with intravenous contrast was obtained, which demonstrated diffuse colonic wall thickening. Her initial laboratory findings were within normal limits, and she was ultimately discharged with a diagnosis of nonspecific colitis. Later that same evening, the patient represented to the referring facility emergency department with worsening of her abdominal pain. Shortly after arrival, she became obtunded, required intubation and had a significant decline in her hemodynamics. Laboratory analysis revealed a hemoglobin of 8.3 g/dL (15.9 g/dL at the time of primary evaluation), a lactic acid of 20 and a pH of 6.8. An emergency CT angiogram was obtained. This revealed large amounts of intra-abdominal fluid consistent with hemoperitoneum that was new from the previous scan. It also demonstrated an arterial enhancing 13 mm central left hepatic lesion that represented a hepatic pseudoaneurysm (Fig. 1). Blood product administration was initiated, and the patient was transferred to our facility for further management.

**Figure 2:**
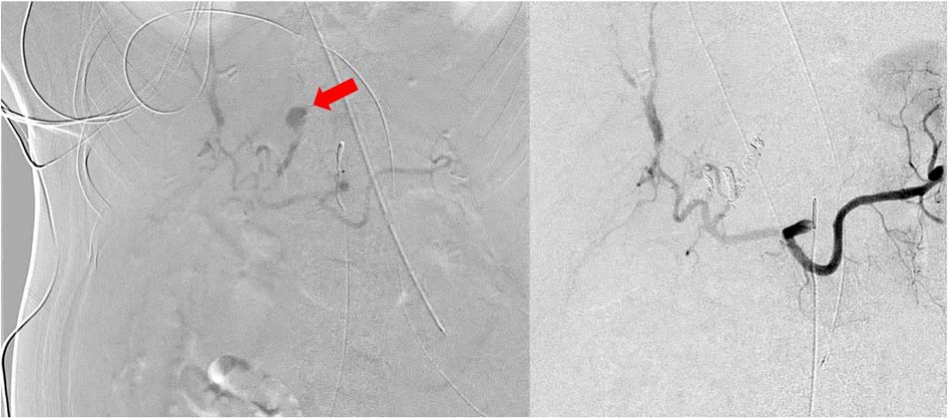
Conventional angiographic images. Left HAP identified on hepatic artery angiogram (left). Following angioembolization, there is no further evidence of bleeding from the liver via celiac artery angiography (right). Red arrow is identifying left HAP.

On arrival, the patient was emergently taken to the IR suite for diagnostic angiography and embolization. Angiography identified active extravasation of three separate branches of the left hepatic artery from pseudoaneurysms (Fig. 2). These were embolized. Along the right hepatic arterial system, there was small aneurysmal dilation but no bleeding. Upon completion of her angiogram, she was transferred back to the ICU in critical condition. There was an initial significant improvement in her hemodynamics and stabilization of her hemoglobin. On post-procedure Day 1, the patient again had worsening hemodynamics with a drop in her hemoglobin. A repeat angiogram was performed by IR, but no active bleeding was identified. The hemoglobin again stabilized. Unfortunately, the patient’s clinical picture continued to deteriorate with development of liver failure, kidney failure and worsening respiratory failure despite maximal medical efforts. The family of the patient ultimately decided to pursue comfort focused measures instead of aggressive life sustaining measures and the patient expired on hospital Day 2.

## DISCUSSION

VAAs are rare but potentially life-threatening. It is estimated that approximately one-fourth of VAAs present with rupture, and of these, 10% prove fatal [6, 7]. With increased use of intra-abdominal imaging, VAAs are being identified more frequently. However, because of the relative rarity of VAAs, the natural history has been difficult to delineate. It is known that visceral pseudoaneurysms have a higher risk of rupture than true aneurysms. Of the VAA, HAA are the second most frequent type [6]. While HAA can be closely monitored if they are asymptomatic, <2 cm in size, or has a growth rate of <0.5 cm/year, the Society of Vascular Surgery recommends treatment of HAP regardless of the cause because of the high propensity for rupture [6]. Once identified, the optimal treatment should be with coil embolization. While surveillance is not recommended for HAP, recommendations for HAA suggest one-time screening angiography of the chest, head and neck in patients with nonatherosclerotic etiologies to rule out concomitant aneurysmal disease. An annual CTA scan to observe patients with asymptomatic HAA is suggested [6].

While HAP is a very rare condition, most patients have predisposing risk factors that leads to its development. Most HAP are because of iatrogenic injury. These patients generally have had a recent procedure, such as cholecystectomy or percutaneous biliary procedure [3, 4]. This patient did not have any significant surgical or procedural history that would account for an iatrogenic cause of her HAP. There are reports of patients with malignancies who are also at increased risk, but this patients AFP, CEA and CA 19-9 were all within normal limits and no tumor or mass was identified on the numerous imaging studies that were performed. There have been few reports in the literature regarding idiopathic hepatic pseudoaneurysm rupture [8]. One patient who did have a rupture HAP presented with a hypertensive urgency, which was thought to contribute to the rupture of this pseudoaneurysm. While our patient did present with initial hypertension (blood pressure 179/85 mmHg), it is unknown if there is a direct correlation between hypertension in our patient and the risk of pseudoaneurysm rupture.

In conclusion, HAP are a rare but potentially life-threatening cause of intraabdominal hemorrhage. Given the risk of rupture and high rate of mortality associated with rupture, aggressive treatment for HAP should be pursued and most often should involve endovascular or angiographic approaches with a surgical approach reserved for a very limited number of cases. Identification of patients who are at the risk of HAP and how best to monitor them is needed to decrease the morbidity and mortality of this rare condition.
